# Auditory brainstem response audiometry in tauopathy mouse model of human Alzheimer's disease

**DOI:** 10.1121/10.0026602

**Published:** 2024-07-09

**Authors:** Kali Burke, Laurel A. Screven, Sergio Vicencio-Jimenez, Amanda M. Lauer

**Affiliations:** 1 Department of Otolaryngology-Head and Neck Surgery, Johns Hopkins University School of Medicine, Baltimore, Maryland 21205, USA; 2 Department of Neuroscience, Johns Hopkins University School of Medicine, Baltimore, Maryland 21205, USA kburke50@jhmi.edu; laurel.screven@nih.gov; svicenc1@jhmi.edu; alauer2@jhmi.edu

## Abstract

Alzheimer's disease (AD) is a progressive neurodegenerative disorder in which changes in hearing sensitivity precede cognitive decline. Despite a well-known link between dementia and hearing loss, few AD model mouse lines have hearing characterized. We screened for hearing loss using auditory brainstem responses (ABR) in young (3–4 months) and aging (9–10 months) mice with a P301S tauopathy (PS19 mice). Compared to wild types, aging PS19 mice did not show accelerated hearing loss but did show latency differences in centrally generated ABR waveform components. These results suggest that tauopathy causes mild central auditory dysfunction in the absence of overt hearing loss.

## 1. Introduction

Alzheimer's disease (AD) is associated with auditory deficits in humans, and recent findings suggest that hearing loss may precede cognitive decline in humans and mice, serving as a potential diagnostic biomarker.^1,2^ Hearing loss is a potential modifiable risk factor that could mitigate or prevent the progression of dementia if identified and treated in at-risk individuals.^3–6^ Developing treatments or strategies to prolong the onset of progressive neurodegenerative diseases requires the careful selection of preclinical animal models that accurately represent different aspects of the heterogeneous human condition. To this end, several human genetic mutations associated with AD have been incorporated into mouse models. Using animal models, the causal role of a particular gene in potentially accelerating the time course of hearing loss and subsequent development of overt AD symptoms can be assessed to identify targets for drug-based intervention.

Many commonly used AD mouse models carry familial AD mutations, including the APP/PS1, 5xFAD, and P301S (PS19) models, resulting in early-onset neuropatholgies. The PS19 transgenic mouse expresses the T34 isoform and four microtubule binding repeats (1N4R) of the tau protein with P301S mutation under the regulatory control of the murine prion promoter. PS19 mice are used to study tau aggregation, tauopathy, and other neuropathologies related to AD (University of Pennsylvania, Philadelphia, PA; JAX 008169^12^) Mice with the P301S mutation have reduced acoustic startle responses and abnormal prepulse inhibition of the startle response compared to wild types, but it is unclear if these effects are related to hearing loss.^7,8^

We evaluated the hearing status of young and aging PS19 and wild type (henceforth WT) mice to see if PS19 transgenic (henceforth PS19) mice exhibited accelerated age-related hearing loss relative to WT mice. Previous studies of APP/PS1 and the 5xFAD transgenic mice suggest that hearing dysfunction occurs before the onset of severe pathology in AD mouse models.^1,9–11^ Identifying which genetic mutations are associated with accelerated hearing loss may provide a biomarker of the underlying neuropathology and clarify the role of hearing loss in disease progression in these familial mutations. Our measurements show similar auditory thresholds in PS19 and WT mice at 3–4 months and 9–10 months, and that no significant age-related hearing loss occurred over this time. This finding is surprising considering the onset of neurofibrillary tangles in the brainstem of these mice by five months of age.^1,12^

## 2. Methods

### 2.1 Animals

Mice were obtained from the Jackson Laboratory (B6;C3-Tg(Prnp-MAPT^*^P301S)PS19Vle/J; RRID:IMSR_JAX:008169). Twenty mice were evaluated longitudinally, ten PS19 [five male (M), five female (F)], and ten WT (five M and five F). Between the 3–4 month and 9–10 month assessments, three PS19 mice died, leaving seven (two M and five F) contributing to that age. All mice were housed in standard filter-top shoebox cages in a quiet satellite facility with access to food and water *ad libitum*, controlled temperature conditions, and a 12 h light-dark cycle (daily light period, 6 a.m. to 6 p.m.). All procedures were approved by the Institutional Animal Care and Use Committee at Johns Hopkins University, and all conditions complied with the Guide for the Care and Use of Laboratory Animals.

### 2.2 Auditory brainstem response (ABR)

We recorded ABRs in both PS19 and WT mice using procedures similar to those described in our previous publications.^15–18^ Mice were anesthetized with an intraperitoneal injection of 100 mg/kg ketamine and 20 mg/kg xylazine and placed on a heating pad to maintain an internal temperature of 37 °C. Subdermal needle electrodes were placed along the vertex and the bulla for a differential recording and a ground electrode was inserted at the hind leg.

Free-field sound stimuli were generated and responses were recorded inside an IAC sound-attenuating recording chamber using Tucker-Davis Technologies (TDT) System III hardware (TDT RZ6 Multi I/O processor) and SigGen/BioSig software (TDT, Alachua, FL). Acoustic stimuli were presented using a TDT magnetic speaker (model MF1; TDT, Alachua, FL). Stimuli consisted of clicks (0.1-ms square wave pulses), and tones (5-ms, 0.5 ms rise/fall times) at frequencies of 8, 12, 16, 24, and 32 kHz presented at a rate of 21/s and with alternating polarity to reduce stimulus artifacts. We calibrated stimuli using a 1/4 in. free-field microphone (PCB model 426BO3, PCB, Depew, NY) placed at the position of the animal's ear during ABR recording experiments (10 cm from the MF1 speaker at 0 degrees azimuth). We presented click and tone stimuli at levels ranging from 90 to 10 dB re 20 *μ*Pa in 10 dB decrements until a threshold was reached. Responses were averaged across 512 stimulus presentations, amplified using a RA4PA Medusa pre-amplifier (TDT, Alachua, FL), recorded at a 12 kHz sampling rate, and filtered using 300 Hz high-pass and 3000 Hz low-pass Butterworth filters.

We defined response thresholds as the intermediate sound level between the lowest stimulus level that evoked a response that was discernable from noise and the level at which no response was observed. For cases where no thresholds were obtained for stimuli presented at the highest intensities, a threshold value of 90 dB re 20 *μ*Pa was used for statistical analyses. Thresholds were determined by visual inspection by two independent experienced observers blinded to the genotype and age of the subjects. The amplitudes and latencies of ABR waves 1–5 evoked by 90 dB re 20 *μ*Pa stimuli were identified using a custom program (described in;^19^ software available at: https://github.com/mattbke63/Auditory-Brainstem-Response-Waveform-Analysis), followed by visual verification of the automatically detected peaks and troughs. Amplitudes were measured as the maximum peak-to-trough voltage of each ABR wave, and latencies were measured as the timing of the maximum voltage peak of each wave relative to the timing of stimulus onset.

### 2.3 Statistical analysis

Linear mixed effects modeling was conducted in R version 4.0.3^20^ using the packages lme4^21^ and emmeans^22^ to evaluate whether the interaction between genotype (PS19 or WT), age (3–4 months or 9–10 months), and stimulus (click, 8, 12, 16, 24, and 32 kHz) could be used to predict the threshold and the amplitude or latency of waves 1–5. Significant comparisons of fixed effects were Tukey adjusted.

## 3. Results and discussion

**Fig. 1.**
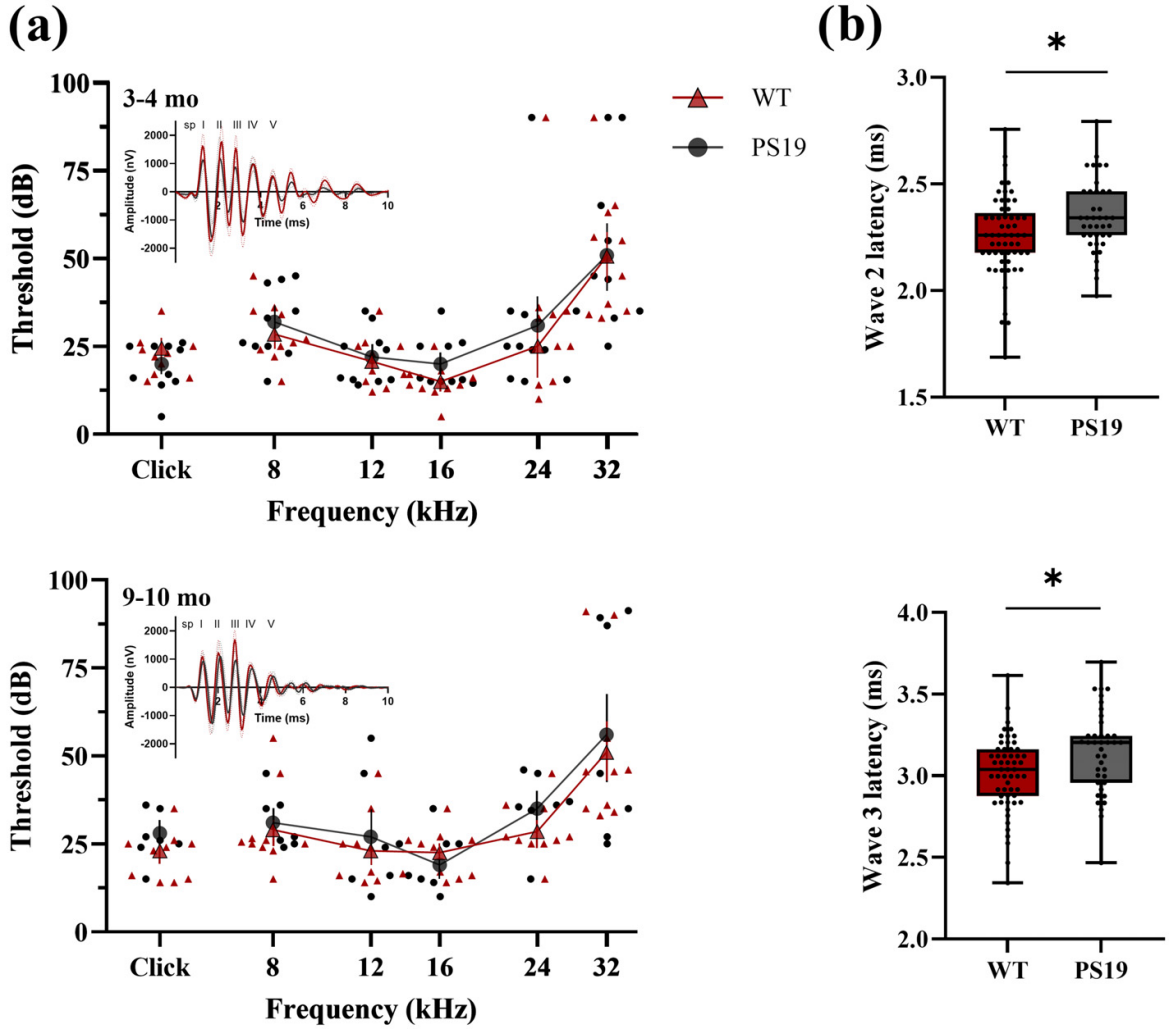
(a) ABR derived audiogram from three to four months (top) and from nine to ten months (bottom) mice that are PS19 (black, *N* = 10) and WT (red, *N* = 10) for five tone frequencies and clicks. Error bars are standard errors of the mean (SEM). Inset example waveforms recorded in response to a 90 dB broadband click from each group are shown in the corner of (a), demonstrating slightly smaller, but nonsignificant, response amplitudes in PS19 mice. (b) Latencies of wave 2 (top) and 3 (bottom) for the nine and ten month WT and PS19 mice. Latencies are significantly delayed (indicated with ^*^) across genotypes at this age. The boxplot represents the 25–75 percentile, error bars are the min and max values, and the line is the median.

Auditory sensitivity of young (3–4 months, prior to the onset of neurofibrillary tangle formation in the brainstem) and aging (9–10 months, aged past the onset of neurofibrillary tangle formation) PS19 and WT mice was measured to determine whether PS19 mice exhibit accelerated age-related hearing loss relative to WT mice. ABR thresholds were similar at 3–4 months and 9–10 months in PS19 and WT mice (Fig. 1). Still, a significant main effect of age was found when the threshold was averaged across stimuli and genotypes (*F* = 4.82, *p* = 0.03) (3–4-month-old = 28.8 dB ± 1.98 dB, 9–10-month-old = 32.2 dB ± 2.09 dB). A significant main effect of frequency occurred, as expected given that hearing sensitivity varies as a function of frequency in general (*F* = 41.54, *p* < 2.2 × 10^−16^). No other significant main effects or interactions were observed. *Post hoc* Tukey analyses revealed a significant difference in threshold between clicks and 24 kHz (*p* = 0.021) and clicks and 32 kHz (*p* < 0.0001), 8 and 16 kHz (*p* = 0.002), 8 and 32 kHz (*p* < 0.0001), 12 and 32 kHz (*p* < 0.0001), 16 and 24 kHz (*p* = 0.0004), 16 and 32 kHz (*p* < 0.0001) and 24 and 32 kHz (*p* < 0.0001). This absence of threshold differences across genotypes suggests that PS19 mice do not have accelerated age-related hearing loss and that reduced hearing sensitivity does not precede the emergence of neuropathology in this strain.

Detailed wave morphology analysis of the ABR response to 90 dB stimuli was also conducted. The amplitude of wave 3 showed a significant interaction between genotype and age (*F* = 7.66, *p* = 0.006), with a significant difference only for the PS19 mice comparing 3–4 months old and 9–10-month-old mice (*p* = 0.0001). Wave 4 also showed a significant interaction between genotype and age (*F* = 4.83, *p* = 0.029) but there were no significant *post hoc* comparisons for this interaction. No other waves showed significant main effects or interactions with genotype for the response amplitudes. There was a significant interaction between genotype and age (*F* = 9.11, *p* = 0.0029) and genotype and stimulus (*F* = 2.41, *p* = 0.038) for the wave 2 latencies. *Post hoc* analyses revealed that the latency of wave 2 was significantly different between WT and PS19 mice at 9–10 months (*p* = 0.04), with PS19 mice having a significantly delayed latency of wave 2 relative to WTs across frequencies (WT = 2.25 ± 0.0301 PS19 = 2.34 ± 0.0346) (Panel B, top). Within PS19 mice there was a significant change in the latency of wave 2 from 3–4 months to 9–10 months (*p* = 0.004). There were significant interactions between genotype and age (*F* = 5.98, *p =* 0.015) and genotype and stimulus (*F* = 3.35, *p* = 0.006) for wave 3 latencies. *Post hoc* analyses revealed that the latency of wave 3 significantly differed between WT and PS19 for 16 kHz tones (*p* = 0.002) (WT = 2.92 ± 0.0558, PS19 = 3.17 ± 0.0578) and at 9–10 months (*p* = 0.017) (WT = 3.01 ± 0.0375, PS19 = 3.15 ± 0.0445) (Panel B, bottom). For the *post hoc* comparisons of genotype and stimulus for both wave 2 and wave 3, there were several significant differences across stimuli within a genotype but no significant differences within stimulus across genotype (see supplementary material for all significant *post hoc* comparisons).

The lack of substantial differences in ABR thresholds between groups suggests that the peripheral auditory system is mainly intact in the PS19 mice, as has been reported in a postmortem human temporal bone study of a small sample of humans with AD.^13^ The ABR is dominated by the ascending auditory tract, and it is hypothesized that wave 2 is driven primarily by globular bushy cells of the cochlear nucleus, and wave 3 is driven by the bushy cell targets in the superior olivary complex in mice.^14,15^ Delays in waves 2 and 3 broadly across frequencies suggest problems with how sound signals are transmitted from the ear to the brainstem or within the brainstem. It is possible that the changes in latency, but not amplitude, reflect synaptic delays, demyelination, or loss of some population of auditory brainstem neurons that is severe enough to reduce the speed of transmission within the brainstem, but not severe enough to substantially diminish the response. It is important to interpret these results with caution given that the changes in latency are pooled across stimulus/frequency as this factor also heavily influences the latency of the response (see the supplementary material for full list of significant comparisons not specific to genotype). Further, it is surprising that delays exhibited in waves 2 and 3 are not enough to persist to significant delays in the transmission of more central waves including waves 4 and 5. Compensatory mechanisms in the central auditory pathway may help to maintain the timing of waves 4 and 5. While synaptic delays or demyelination might affect the initial drivers of auditory processing reflected by earlier waves, more central structures may adapt to preserve overall transmission times, preventing significant delays in later waves. While no threshold differences were observed between genotypes, this study suggests that changes in the morphology of the ABR reflect subtle declines in central auditory system function in the PS19 mouse model of tauopathy.

## 4. Conclusion

In conclusion, PS19 and WT mice have similar auditory sensitivity at 3–4 and 9–10 months of age despite prior evidence suggesting accelerated age-related hearing loss in AD mice by 9–10 months. Sub-threshold differences between genotypes emerging at wave 2 of the ABR suggest differences in the speed of transmission in the central but not the peripheral auditory system, though this was not enough to reduce auditory sensitivity in the ABR measure. This study adds to an existing body of work attempting to understand the impact of various human Alzheimer's mutations on auditory sensitivity in mouse models. Importantly, we want to bring attention to the need for future studies that attempt to reconcile discrepancies within and across AD models to draw parallels more accurately between human and model organism manifestations of AD.

## Supplementary Material

See supplementary material for mean amplitudes and latencies for both age groups and genotypes (Supp 1), output from statistical models for threshold, amplitude, and latency (Supp 2), and all significant post-hoc comparisons (Supp 3).

## Data Availability

The data that support the findings of this study will be publicly available on the Johns Hopkins University data repository following publication of the manuscript. Interested parties may contact the authors for additional data inquiries.
